# The impact of coronavirus disease 2019 on head and neck cancer services: a UK tertiary centre study

**DOI:** 10.1017/S0022215120001735

**Published:** 2020-08-06

**Authors:** R Taylor, E Omakobia, S Sood, R J Glore

**Affiliations:** ENT Department, Bradford Royal Infirmary, Bradford Teaching Hospitals NHS Foundation Trust, UK

**Keywords:** COVID-19, Coronavirus, Retrospective Studies, Laryngopharyngeal Reflux, Head And Neck Neoplasms

## Abstract

**Background:**

The coronavirus disease 2019 pandemic has necessitated almost exclusive National Health Service focus on emergency work and cancer care. There are concerns that increased hospital and community pressures will lead to decreased referrals and worse outcomes for head and neck cancer patients.

**Method:**

This is a retrospective review of all cases referred for suspected head and neck cancer to our institution in January and April 2020.

**Results:**

There was a 55 per cent decrease in referrals but diagnostic yield rose from 2.9 per cent in January to 8.06 per cent in April. In both months, 100 per cent of patients met the 31- and 62-day targets, with similar 14-day wait time success (97.83 per cent for January *vs* 98.33 per cent for April). Referrals for laryngopharyngeal reflux rose from 27.5 per cent to 41.9 per cent. Referrals for those aged over 60 years fell from 42 per cent to 26 per cent.

**Conclusion:**

It is suggested that further research be conducted into the reasons why fewer patients were referred, particularly elderly patients, and why laryngopharyngeal reflux is so prevalent in fast-track referrals.

## Introduction

In December 2019, several cases of pneumonia of unknown aetiology were identified in the city of Wuhan, China. The cause was identified as severe acute respiratory syndrome coronavirus-2 (SARS-CoV-2), a novel coronavirus strain that causes coronavirus disease 2019 (Covid-19) and is suspected to be of zoonotic origin.^1^ The SARS-CoV-2 strain is highly contagious; by 13th June 2020, it had caused 7.6 million confirmed infections worldwide and 426 000 related deaths.^2^

In order to cope with the expected influx of patients with Covid-19, the National Health Service (NHS) had to fundamentally change the way it functions so as to narrow its focus on emergency care, including Covid-19 and cancer care. In order to achieve this, NHS hospitals across the UK were instructed to cancel elective work across all specialties, thereby freeing bed space and allowing the redeployment of staff to areas of need.^3^ In the community, general practice had to rapidly adapt to a sudden change from face-to-face patient reviews to remote consultations via telephone and video. Patients aged over 70 years or those with underlying health conditions were advised to shield at home, to decrease the risk of becoming infected with Covid-19, as the mortality rate for this group is 15 per cent.^4^

The changes to the NHS and the increased anxiety surrounding patients being exposed to SARS-CoV-2 in healthcare settings are barriers to patients seeking medical care. There is concern that an increase in barriers to healthcare will be reflected in decreased referrals to cancer services and potentially missed diagnoses. In addition to decreased referrals, there are further concerns that cancer services themselves may be affected, with decreased staff availability because of self-isolation, Covid-19 infection or shielding. Further compounding this problem is staff redeployment to different areas of the hospital, limiting clinic numbers and the availability of radiological investigations.

This audit of a busy tertiary centre head and neck fast-track referral service assessed how these multiple factors affected head and neck cancer referrals by comparing practice in January 2020 to April 2020. The study also assessed whether the fast-track service at our institution maintained function in line with UK head and neck cancer multidisciplinary team (MDT) guidelines during the Covid-19 pandemic.

## Materials and methods

We conducted a retrospective review of all cases referred for suspected head and neck cancer to the ENT fast-track clinic at our institution during the months of January and April 2020. Referral details were obtained from our electronic referrals system. Data on demographics, investigations and management of patients were collected from our electronic patient record system. Practice was compared against the UK head and neck cancer MDT guidelines.

## Results

### Referrals and cancer diagnoses

There was a 55 per cent decrease in referrals between January and April 2020 (Table 1), with the average wait time for first review remaining stable (6.3 days *vs* 7.29 days). The proportion of patients being seen within 14 days from general practitioner referral, as per MDT guidelines, remained high in both months (97.83 per cent *vs* 98.33 per cent).

**Table 1.** Overview of referrals to fast-track clinic

Parameter	January 2020	April 2020
Referrals (*n*)	138	62
Average wait for 1st review (days)	6.3	7.29
Seen within 14 days (%)	97.83	98.33
Discharged at 1st review (%)	63.04	75.8
Diagnosed malignancies (%)	2.9	8.06

At first review, a significant proportion of patients were discharged without any further investigation for malignancy in both months, with 63.04 per cent of cases in January, rising to 75.8 per cent in April. Whilst there was a decrease in the proportion of patients referred for suspected malignancy, there was a rise in the proportion of patients who had a malignancy diagnosed (2.9 per cent in January *vs* 8.06 per cent in April).

Despite the low yield of cancer diagnoses, 100 per cent of patients in both January and April met both the 31-day and 62-day UK cancer waiting time targets.

### Common diagnoses

**Fig. 1.**
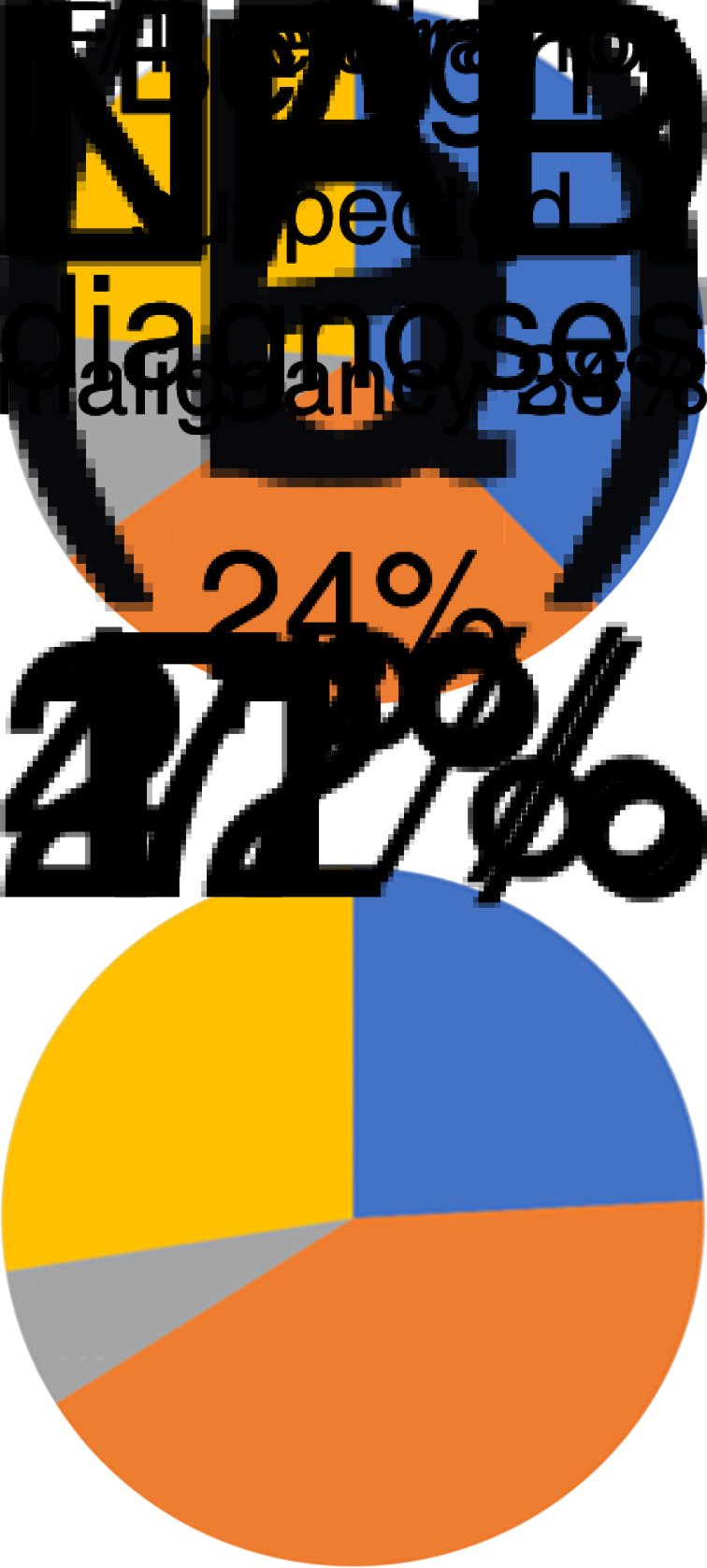
Comparison of outcomes from first review for (a) January and (b) April 2020 referrals. F/T = fast-track; LPR = laryngopharyngeal reflux; NAD = no abnormality detected

The most common diagnosis for patients who were seen was laryngopharyngeal reflux. In January, this accounted for 27.5 per cent of total referrals, rising to 41.9 per cent in April (Figure 1). The proportion of patients diagnosed with other benign diagnoses remained stable (24 per cent *vs* 27 per cent). In addition, the proportion of patients with no pathology on review remained similar, with 11 per cent in January compared to 7 per cent in April.

### Demographics

**Fig. 2.**
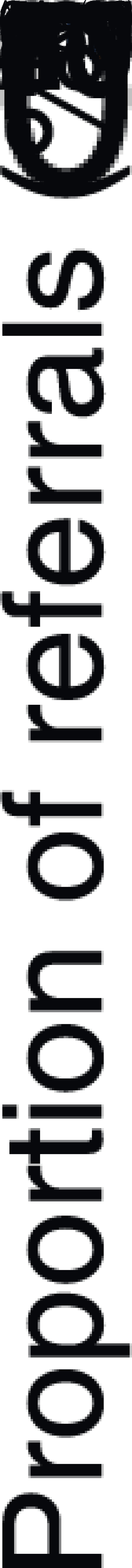
Comparison of the proportion of referrals by age group in January and April 2020.

Analysis of the demographics showed that the change in referral pattern during April was not evenly split by sex and age. In January, males accounted for 47 per cent of referrals, whereas this figure dropped to 40 per cent in April. There was a more marked variation in referrals when analysed by age group; the largest decrease was noted in the late-middle-aged to elderly population, with referrals for patients aged 60 years or more falling from 42 per cent of total referrals in January to 26 per cent in April (Figure 2).

### Imaging investigations

Of those patients referred for a suspicious neck lump, there was a reduction of 55 per cent in total referrals (11 *vs* 5 referrals). Ultrasound scanning remained the most common investigation; 91 per cent of patients referred in January had an ultrasound scan, with an average wait for the ultrasound scan from first review of 5.7 days. This compared to 60 per cent of referrals in April receiving an ultrasound scan, with an average wait time of 5.3 days. There was a rise in cross-sectional imaging, with 9 per cent of patients in January undergoing magnetic resonance imaging (MRI) compared to 20 per cent of patients in April, with an average wait time of 4 days in January and 6 days in April. Diagnostic yield for malignancy increased from 18 per cent of patients referred with a suspicious neck lump in January to 40 per cent in April.

### Thyroid malignancy

The most marked reduction in referrals was for potential thyroid malignancy, with an 88 per cent reduction from January to April. When assessing against British Thyroid Association guidelines, 100 per cent of referrals had at least one ‘red flag’ symptom in April, compared to 65 per cent of referrals in January. However, the proportion of patients who underwent a thyroid function test at their general practitioner fell from 41 per cent in January to 0 per cent in April.

All patients were investigated with an ultrasound scan, with a 7.5-day wait in January falling to a 1-day wait in April. Sixty-five per cent of ultrasound scan reports stated an ultrasound ‘U grade’ classification of thyroid nodules (as per British Thyroid Association guidelines) in January, falling to 50 per cent in April. Fine needle aspiration for cytology was performed in 12 per cent of patients in January compared to 50 per cent in April, with a diagnostic yield for malignancy rising from 5.88 per cent to 50 per cent of referrals from January to April.

## Discussion

Our results showed that the expected drop in referrals to our head and neck fast-track service amounted to 55 per cent. This reduction in referrals could be the result of a wide range of factors. For instance, it is likely that patients did not seek medical attention in order to avoid healthcare settings where the risk of Covid-19 infection is likely to be higher, or because they were shielding. It is also possible that the increased use of remote reviews only by general practitioners decreased the likelihood of patients wishing to contact them, which removed the potential for incidental findings on clinical examination.

Analysis of referrals by age group showed that the biggest drop in referrals was seen in the over-60 years age group, who are the most likely patients to be shielding because of age and co-morbidities. This is particularly concerning given the peak incidence for head and neck cancers in the 70–74 years age group.^5^

Breakdown of referrals by sex showed a small decrease in male referrals, from 47 to 40 per cent. This could be reflective of less health-seeking behaviour in males in general, which may be exacerbated during a pandemic. However, males are almost three times as likely to develop head and neck cancer.^5^ Further research is needed to determine which factors were most prominent in causing a decrease in referrals.

It was suspected that a decrease in referrals would lead to an increased yield of diagnosed malignancies associated with a decrease in ‘worried well’ patients presenting. This is reflected in the overall malignancy rate, which rose from 2.9 per cent to 8.1 per cent. However, there was also an unexpected increase in benign disease being diagnosed at first review. Analysis of outcomes after the first review showed a decreased proportion of patients being investigated for malignancy, falling from 38 per cent to 24 per cent. This may reflect a higher threshold for investigating patients in order to prevent those with a very low risk of malignancy being unnecessarily exposed to potential Covid-19 in hospital. This practice is likely to have been partly supported by the ENT UK Head and Neck Cancer Telephone Triage service tool, which risk stratifies patients into high and low risk groups.^6^

The increase in benign diagnosis at first review is particularly marked for the diagnosis of laryngopharyngeal reflux, with 42 per cent of patients being diagnosed with laryngopharyngeal reflux in April 2020 compared to 27.5 per cent in January 2020. Possible causes for this include: anxiety and stress caused by the pandemic, exacerbating reflux symptoms; or patients being assessed remotely prior to referral and consequently being more likely to be referred than if they had attended a face-to-face consultation. Laryngopharyngeal reflux continues to be a large burden on head and neck cancer services, and further research is required to assess why this is.

There was a 55 per cent decrease in the number of patients referred with suspicious neck lumps. This decrease is greater than expected given that neck lumps are a ‘red flag’ presentation of possible head and neck cancer, and they are recognised by most patients as a finding that needs to be reviewed by a medical practitioner. This likely represents a decrease in health-seeking behaviour for the reasons outlined above regarding attempts to reduce the risk of potential Covid-19 exposure. There was also a decrease in patients receiving an ultrasound scan for their suspicious neck lump, falling from 91 per cent to 60 per cent. The average wait for ultrasound scanning remained similar (5.7 days *vs* 5.3 days), which may reflect the cancellation of routine imaging decreasing the wait time. The decrease in ultrasound scans performed, coupled with an increase in MRI from 9 per cent to 20 per cent, could be the result of more widespread use of telephone consultations, which may have increased diagnostic uncertainty and therefore increased requests for cross-sectional imaging.

• There has been a large decrease in fast-track referrals during the coronavirus disease 2019 pandemic, particularly in the elderly population
• Head and neck cancer services at our institution continue to meet 14-, 31- and 62-day target times, despite changes in services
• There has been an increased diagnostic yield of malignancies
• There has been a large rise in referrals for laryngopharyngeal reflux disease
• More research is required to establish why there has been a large decrease in referral of elderly patients

Suspected thyroid cancer showed the largest decrease in referrals of any single pathology, with an 88 per cent reduction. There was a noticeable increase in the proportion of patients with at least one red flag symptom as per British Thyroid Association guidelines, rising from 65 per cent to 100 per cent. The decrease in referrals for suspected thyroid malignancy could be the result of the increased use of telephone consultations by general practitioners, precluding clinical examination. Additionally, there may have been reduced patient willingness to attend hospital, resulting in less patient pressure for a referral.

The British Thyroid Association guidelines require general practitioners to check thyroid function test results prior to referral to secondary care. This was performed for 41 per cent of referrals in January and for no patients at all in April, which represents the closure of general practitioner practices for face-to-face consultations, including phlebotomy, in the pandemic. There is no clear reason why 59 per cent of patients could not have thyroid function tests in January. It does perhaps suggest that further education should be provided to general practitioners regarding the British Thyroid Association guidelines for secondary care referral for suspected thyroid malignancy.

As per British Thyroid Association guidelines, all patients in both months had an ultrasound scan of their thyroid, with a ‘U’ grade reported (as per British Thyroid Association guidelines) for 65 per cent of scans in January and 50 per cent in April. The most common reason for not reporting a U grade was that normal thyroid tissue was not documented as ‘U1’.

The decrease in referrals and increase in proportion of patients with red flag symptoms led to an increase in yield for diagnosing thyroid malignancy, rising from 5.88 per cent to 50 per cent. This suggests that if British Thyroid Association guidelines are followed, there would potentially be fewer patients referred with benign or no disease.

## Conclusion

The results of our audit show that despite the Covid-19 pandemic, the fast-track head and neck service at our institution met the 14-day, 31-day and 62-day target times in the month of April 2020, when compared to pre-pandemic data. There has not been a drop in the overall number of malignancies diagnosed, as may have been expected despite the significant fall in referrals, particularly among the elderly.

Future audits will be required to see if there is a rise in malignant diagnoses in the coming months or whether those patients not referred represent the ‘worried well’. With an easing of lockdown restrictions across the UK, it is expected that health-seeking behaviours will begin to normalise and this may cause a spike in referrals; with hospitals still readjusting following Covid-19, this may make it difficult for the two-week wait guidance to be adhered to in the near future.

A final key finding of this audit is the markedly low yield of diagnosed malignancies, both before and during the pandemic, with huge numbers of laryngopharyngeal reflux cases. This mirrors other studies in the literature.^7^ We recommend that further studies investigate why these patients are being referred so commonly to head and neck cancer services, in order to prevent unnecessary patient anxiety and to allow more time for patients with malignancy to be reviewed.
